# Severe *Salmonella* spp. or *Campylobacter* spp. Infection and the Risk of Biliary Tract Cancer: A Population-Based Study

**DOI:** 10.3390/cancers12113348

**Published:** 2020-11-12

**Authors:** Elise de Savornin Lohman, Janneke Duijster, Bas Groot Koerkamp, Rachel van der Post, Eelco Franz, Lapo Mughini Gras, Philip de Reuver

**Affiliations:** 1 Department of Surgery, Radboud University Medical Center, P.O. Box 9101, 6500 Nijmegen, The Netherlands; elise.desavorninlohman@radboudumc.nl; 2 National Institute for Public Health and the Environment, P.O. Box 1, 3720 Bilthoven, The Netherlands; janneke.duijster@rivm.nl (J.D.); eelco.franz@rivm.nl (E.F.); lapo.mughini.gras@rivm.nl (L.M.G.); 3 Department of Surgery, Erasmus University Medical Center (ErasmusMC), P.O. Box 2040, 3000 Rotterdam, The Netherlands; b.grootkoerkamp@erasmusmc.nl; 4 Department of Pathology, Radboud University Medical Center, P.O. Box 9101, 6500 Nijmegen, The Netherlands; chella.vanderpost@radboudumc.nl; 5 Institute for Risk Assessment Sciences, Utrecht University, P.O. Box 80125, 3508 Utrecht, The Netherlands

**Keywords:** bacterial infection, cancer, epidemiology, risk factor

## Abstract

**Simple Summary:**

Although it is known that bacterial infection may increase risk of cancer, the relationship between certain infections and cancer remains ill-quantified. To identify potential risk factors, this study compared the incidence of biliary tract cancer (BTC) in patients with *Salmonella* spp. or *Campylobacter* spp. infection to the general population in a large Western cohort of 16,252 salmonellosis and 27,668 campylobacteriosis patients. Standardized relative incidence ratio for BTC was 1.53 (95% CI 0.70–2.91) in salmonellosis patients and 0.97 (95% CI 0.39–2.00) in campylobacteriosis patients. Patients with *Salmonella* spp. infection and BTC were significantly younger than BTC patients without *Salmonella* spp. infection. Potentially, the study was underpowered to detect differences in cancer incidence, or cancer etiology in Western patients differs from those in non-Western countries and instead of bacterial infection, other factors contribute to cancer risk. Better understanding of cancer etiology is needed to identify risk factors and facilitate screening and early detection of cancer patients.

**Abstract:**

*Salmonella* spp. infection has shown to have oncogenic transformative effects and thereby increases the risk of certain cancers. For *Campylobacter* spp., similar effects have been demonstrated. Risk factor identification may allow for timely diagnosis and preventive treatment. To substantiate the oncogenic potential of *Salmonella* and *Campylobacter* spp., this study compared the incidence of extrahepatic biliary tract cancer (BTC) in patients with diagnosed *Salmonella* or *Campylobacter* spp. infection with BTC incidence in the Netherlands. National infectious diseases surveillance records of patients diagnosed with a laboratory-confirmed *Salmonella* or *Campylobacter* spp. infection during 1999–2016 were linked to the Netherlands Cancer Registry. Incidence of BTC in *Salmonella* and *Campylobacter* spp. patients was compared to the incidence of BTC in the general population using Standardized Incidence Ratios (SIRs). In total, 16,252 patients were diagnosed with *Salmonella* spp. and 27,668 with *Campylobacter* spp. infection. Nine patients developed BTC at a median of 46 months (13–67) after *Salmonella* spp. infection and seven at a median of 60 months (18–138) after *Campylobacter* spp. infection. SIR of BTC in salmonellosis patients was 1.53 (95% CI 0.70–2.91). In patients aged <60 years, the SIR was 1.74 (95% CI 0.36–5.04). For campylobacteriosis patients, the SIR was 0.97 (95% CI 0.39–2.00). Even though *Salmonella* or *Campylobacter* spp. infection was not significantly associated with increased BTC risk in this cohort, it remains extremely important to study potential risk factors for cancer to facilitate screening and ultimately improve prognosis of cancer patients.

## 1. Introduction

Biliary tract cancers (BTC) are rare malignancies of the distal and proximal bile ducts, the gallbladder and the cystic duct. Despite significant improvement in the overall survival of cancer patients, 5-year survival of patients with extrahepatic biliary tract cancer (i.e., gallbladder cancer, proximal and distal cholangiocarcinoma) is still only 10% [1,2,3]. Currently, radical surgery is the only curative treatment available. Unfortunately, surgery is not an option in the majority of patients, because BTC frequently goes undetected until the disease has progressed to an advanced, unresectable stage [4,5].

Geography appears to be the primary risk factor for the development of non-intrahepatic BTC, and as a result, incidence rates vary significantly per region. For example, gallbladder cancer (GBC) incidence ranges from 0.9/100,000 women in the Netherlands to 35/100,000 women in Chile [6,7]. Other risk factors for BTC include age, parasitic infections, congenital malformations of the biliary tract and primary sclerosing cholangitis, and sex [8]. However, most patients with BTC do not have any of the known risk factors apart from age [9]. Screening for and detection of risk factors in addition to geography and age could lead to significantly faster detection of BTC and a subsequent improvement in survival.

An estimated 20% of the global cancer burden can be attributed to infectious diseases [10]. The association between viral infections, such as human papilloma virus, hepatitis B and C and certain forms of cancer, has been well-established [11,12]. This knowledge has led to the implementation of successful targeted treatment and screening programs that can facilitate prevention and early detection of these cancers and improve survival, such as the Dutch national program for cervical cancer [13]. Although less studied, bacteria also have oncogenic potential and thereby increase the risk of cancer [14]. The primary example is *Helicobacter pylori* infection, which increases the risk of gastric cancer through the secretion of toxins that mediate cell signaling, as well as chronic inflammation [15]. Similarly, *Salmonella* spp. enforce bacterial uptake by manipulating host cell signaling pathways. Specifically, host AKT and ERK pathways are activated. Both pathways are active in many cancers and are an essential step in the malignant transformation of pre-transformed cells [16]. *Salmonella* spp. infection is common and represents a known risk factor for gallbladder and colon cancers, with the former pertaining specifically to *Salmonella typhi*, the agent of typhoid fever, and the latter to non-typhoidal *Salmonellae* [16,17]. However, the role of non-typhoidal *Salmonella* has not yet been investigated for other biliary cancers. *Campylobacter* spp. is another frequently-occurring gastrointestinal infection able to promote colon tumorigenesis by producing cytolethal distending toxins and is more frequently present in the microbiome of colorectal cancer patients, although a causal relationship between colorectal cancer and *Campylobacter* spp. infection has not been demonstrated [18,19,20,21].

*Salmonella* spp. is known to cause chronic inflammation of the bile ducts and to produce toxins with carcinogenic potential, which may lead to cancer of the extrahepatic biliary tract [22]. After an outbreak of *Salmonella typhi* in 1964, researchers found that the risk of biliary tract cancer was increased by 164 times in carriers compared to non-carriers [23]. Although non-typhoidal *Salmonella* has been associated with the development of colon cancer, its role has not been specifically investigated in biliary tract cancers other than gallbladder cancer [17]. *Campylobacter* spp. is found in abundance in the biliary microbiome of patients with BTC [24]. The potential association with non-typhoidal *Salmonella* or *Campylobacter* spp. and BTC has not been studied in large cohorts due to the rarity of BTC, especially in Western populations. In case an association is found, targeted screening for BTC in *Salmonella* spp. and *Campylobacter* spp. patients might be considered. To assess whether infection with non-typhoidal *Salmonella* or *Campylobacter* spp. is a risk factor for BTC, this study compares the incidence of BTC in patients with a registered non-typhoidal *Salmonella* or *Campylobacter* spp. infection in the past to the incidence of BTC in a Western–European population.

## 2. Results

### 2.1. Cohort Characteristics

**Figure 1:**
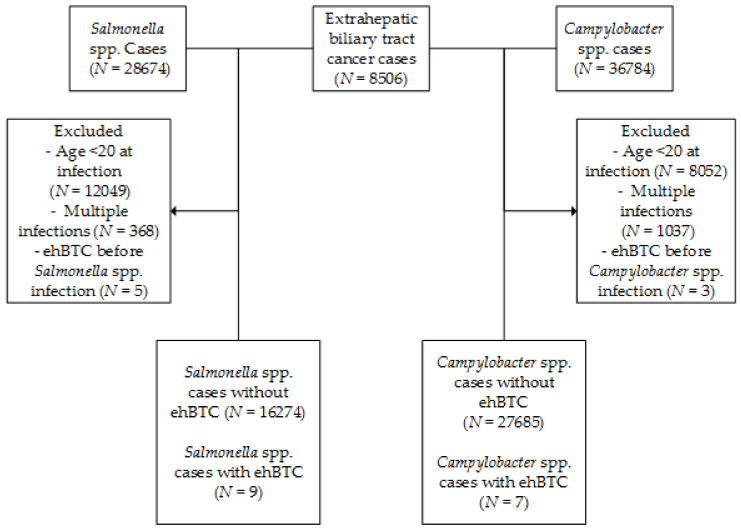
Cohort selection. ehBTC = extrahepatic biliary tract cancer.

The final cohort consisted of 16,283 *Salmonella* spp. patients (reported between 1999–2016), 27,692 *Campylobacter* spp. patients (reported between 2002–2016) and 8506 patients with BTC (Figure 1). After linkage, nine *Salmonella* spp. patients and seven *Campylobacter* spp. patients were diagnosed with BTC ≥1 year after infection.

Baseline characteristics of the cohorts are provided in Table 1 (Salmonellosis and Campylobacteriosis) and Table 2 (BTC). Median age at infection was 48.9 years (IQR: 30.0–66.0) for salmonellosis patients (66.5%, <60 years) and 48.5 years (IQR: 31.3–62.4) for campylobacterosis patients (70.8%, <60 years). Median follow-up after infection was 7 years (IQR 3–12) in salmonellosis patients and 5 years (IQR 3–9) in campylobacterosis patients. Median age at diagnosis was 73 years (IQR 64–80) in BTC patients. Median follow-up time from diagnosis to death or end of study in in BTC patients was 55 months.

### 2.2. Patients with Salmonella spp. Infection and BTC

Nine salmonellosis patients were diagnosed with BTC ≥1 year after salmonellosis diagnosis (Table 3). Mean time to BTC diagnosis was 47 months (range 13–81). Three of nine (33%) salmonellosis patients were ≤50 years old at time of BTC diagnosis, as opposed to the general BTC population, in which only 5.0% of patients were ≤50 years old at time of BTC diagnosis (*p* < 0.001). Four cases were diagnosed with *S. enteritidis*, three with *S. typhimurium*, and two with other *Salmonella* serovars. Eight patients had an enteric infection, one had an invasive (bloodstream) infection. Two patients had a distal cholangiocarcinoma, one patient had a proximal cholangiocarcinoma, one patient had gallbladder cancer, and five had BTC NOS (not otherwise specified).

### 2.3. Patients with Campylobacter spp. Infection and BTC

Seven campylobacteriosis patients were diagnosed with BTC ≥1 year after diagnosis (Table 4). Mean time to BTC diagnosis was 60.6 months (range 18–138). All patients were >50 years of age at time of BTC diagnosis. Five patients had a proximal cholangiocarcinoma and two patients had gallbladder cancer.

### 2.4. Risk of BTC After Salmonella spp. or Campylobacter spp. Infection

The SIR of BTC among the salmonellosis patients (compared to the general population) was 1.53 (95% CI 0.70–2.91, Table 5) and the absolute risk was 0.05%. Subgroup analysis in patients <60 years of age demonstrated that the SIR in this group was 1.72 (CI 0.36–5.04). Subgroup analysis according to gender revealed similar findings. In campylobacteriosis patients, the SIR was 0.97 (95% CI 0.39–2.00, Table 5) and the absolute risk was 0.03%. Subgroup analyses stratified according to gender and age revealed similar results.

## 3. Discussion

This study assessed whether *Salmonella* spp. or *Campylobacter* spp. infection represents a significant risk factor for BTC by comparing the incidence of BTC in patients with a history of *Salmonella* spp. or *Campylobacter* spp. infection to the (age-, gender- and calendar year-matched) incidence of BTC in the general Dutch population. Additionally, age and gender effects on the association between *Salmonella* spp. or *Campylobacter* spp. infection and BTC were investigated. No significant increase in BTC occurrence in patients who had experienced a severe *Salmonella* spp. or *Campylobacter* spp. infection was observed.

The relatively low number of *Salmonella* spp. (and *Campylobacter* spp.) infections linked to the (already rare) BTC patients found in this study was the main limitation for statistical significance, as considerable uncertainty was introduced in the estimates by such low number of outcome events. The upper limit of the SIR for BTC in salmonellosis patients was 2.7, which implies that a clinically significant effect may be present, but the study is simply insufficiently powered to detect its presence. This issue is, however, not unique to this study alone, but rather affects all studies investigating rare diseases. Experts increasingly recognize that some evidence, although maybe imprecise, may be better than no evidence at all [25].

In countries where typhoid fever is still endemic, such as the Indian subcontinent and some parts of South America, multiple epidemiological studies have shown an increased risk for the development of BTC and especially gallbladder cancer. Besides chronic infection, an increased risk of gallstones in these populations, a higher incidence of obesity, and potential environmental pollution have been mentioned as potentially contributing to this phenomenon [22]. However, none of these factors (apart from gallstones and gallbladder cancer, which is not unique to these countries) show an extremely high correlation with the incidence of BTC. On the other hand, researchers have demonstrated a clear association with chronic *S. typhi* infection and the development of gallbladder cancer in these countries [26]. In contrast, a Chinese study investigating the correlation between chronic infection with *S. typhi* and biliary tract cancer failed to find a significant association due to a very low occurrence of such infection [27]. One may argue that association does not equal causation and that in areas with endemic typhoid fever and high rates of gallbladder cancer, other factors might be at play as well. However, even in Western countries with typically extremely low incidence of *S. typhi* infection (as typhoid fever has been eradicated in most Western countries thanks to modern sanitation), after large outbreaks of typhoid fever, an increase in number of BTC diagnoses was observed [23].

This paper focusses primarily on the incidence of BTC in non-typhoidal *Salmonella.* We hypothesized that, similar to gallbladder cancer, the increased incidence of BTC after typhoidal *Salmonella* infection would translate to increased BTC risk in non-typhoidal *Salmonella* [28]. The lack of significant correlation in non-typhoid *Salmonella* infection may be attributed to the fact that non-typhoid *Salmonella* strains are less likely to cause chronic infection and thus have lower oncogenic potential compared to their typhoid counterparts [29].

Remarkably, one third of the patients with both *Salmonella* spp. infection and BTC were under 50 years of age at time of BTC diagnosis. This proportion was significantly higher than in the general BTC population, in which only 5% is aged 50 years or younger [30]. Because the risk of BTC increases exponentially with age, we performed a subgroup analysis in all patients aged <60. Although this subgroup analysis also failed to reach significance due to the even lower numbers, the relatively high proportion of young patients suggests that *Salmonella* spp. infection at a young age might contribute to the risk of developing BTC later in life. Possibly, patients who acquire a *Salmonella* spp. infection at the age of 70 or older may die from other diseases before they develop BTC and are thus less well-represented. The median time between *Salmonella* spp. infection and BTC diagnosis was 4 years. This finding implies that the potential oncogenic effect of *Salmonella* spp. results in malignant transformation of epithelial cells in a relatively short timeframe and is concurrent with other studies [17]. Another explanation may be that patients with inflammatory bowel disease (IBD) are at a higher risk for developing a serious *Salmonella* spp. infection. Since IBD often has an onset in early adulthood and is also a potential independent risk factor for the development of BTC, it is possible that this difference in age can be explained by the fact that the patients with *Salmonella* spp. infection also had IBD and therefore were at greater risk for developing BTC at a younger age [31].

No tendency towards increased BTC incidence after *Campylobacter* spp. infection was seen in this study. *Campylobacter* spp. and *Salmonella* spp. bacteria both release the genotoxic protein cytolethal distending toxin (CDT). However, whereas *Salmonella* spp. is linked to the development of BTC by overexpression of C-myc in tissue samples, *Campylobacter* spp. is not [16]. Differences in bacterial mechanisms, specifically concerning the alteration of host cell signaling pathways during invasion, may account for differences in oncogenic potential between the two species.

Molecular characterization of cancers and subsequent personalization of therapy is a prime topic in current oncological research. Although the genomic landscape of BTC is incredibly diverse, multiple preclinical and clinical models show that BTC development may be associated with the alteration of several actionable genes. A particular example is the overexpression of cyclophilin-A in patients with liver-fluke-associated cholangiocarcinoma [32,33]. Identification of inflammation-associated driving mutations is an important topic as it has implications for both risk profiling and potential personalized treatment. Although molecular profiling of patients with salmonellosis and BTC was outside of the scope of this study, a study in gallbladder cancer has managed to identify the signaling pathway associated with *S. typhi* development and gallbladder cancer [16]. Further research investigating molecular alterations in infected cancer patients is paramount to increase our understanding of tumor cell transformation and cancer development.

The primary limitation of this study is the low number of *Salmonella* spp. and *Campylobacter* spp. infected patients that also developed BTC, leading to a high risk of type-2 error. Typically, patients with *Salmonella* spp. infection in the Netherlands who require medical attention, laboratory diagnosis and reporting to health authorities are severely ill. As most patients with *Salmonella* spp. infection only show mild symptoms, the actual number of *Salmonella* spp. cases in the Netherlands is much higher than reported. It is estimated that close to 1 million inhabitants developed a symptomatic *Salmonella* spp. infection in the Netherlands between 1999–2015, which is 35 times the number of cases included in this study. Campylobacteriosis cases are estimated around 81,000 in the Netherlands annually [34]. As a result, a number of patients with mild and therefore unreported *Salmonella* spp. or *Campylobacter* spp. infection, but with a BTC diagnosis, may have been misclassified and included in the group of BTC patients without (reported) *Salmonella* spp. or *Campylobacter* spp. infection. Since the contribution of these mild infections to the risk of developing BTC is implicitly included in the baseline risk, our results may be considered as very conservative estimates of their true contribution to BTC risk. Moreover, although chronic infections are those mostly implicated in BTC formation, they could not be studied as such in this study because this information (i.e., differentiation between acute and chronic infection) is simply not available in the RIVM data set [35]. Yet, we included all reported infections, and because these infections represent the most severe ones (in terms of magnitude and duration of symptoms) occurring in the population, our analysis implicitly focused on a selection of salmonellosis and campylobacteriosis patients that showed extreme clinical manifestations. Finally, the RIVM registry only contains data on *Salmonella* spp. and *Campylobacter* spp. infection from 1999 onwards and consequently we only had a median follow-up period of 7 years. If, like in pancreatic cancer, the interval between first mutation and cancer development is over 10 years, the study period may have been insufficient to detect a correlation between infection and BTC development [36].

A major strength of this study is the cohort size and nation-wide design. Indeed, it should be acknowledged that the low number of BTC events in our cohort—despite the large surveillance data sets used—reflects mainly the rare occurrence of these tumors. The cohort analyzed in this paper is large and comprehensive, being nation-wide and covering all available years of systematic data collection. Previous studies investigating the role of bacterial infections in the development of BTC have typically drawn from case-control cohorts or small case series. Additionally, to our knowledge, this paper describes the first Western cohort of patients with *Salmonella* spp. or *Campylobacter* spp. infection and BTC [26].

## 4. Materials and Methods

### 4.1. Data Collection and Linkage

Analyses were based on three linked health registries with national coverage. The first registry contains records from laboratory-confirmed human infections with *Salmonella* spp. (from 1999 onwards) and *Campylobacter* spp. (from 2002 onwards) based on the national laboratory surveillance system for gastrointestinal pathogens coordinated by the Dutch National Institute for Public Health and the Environment (RIVM) [35]. The surveillance system has an estimated coverage of the resident Dutch population of 64% for *Salmonella* spp. and 52% for *Campylobacter* spp. infection [37]. The second registry consisted of histopathological records provided by the automated pathological archive, the nation-wide network of histopathology and cytology in the Netherlands (PALGA) [38]. The third registry was the Netherlands Cancer Registry (NCR) [39], which contains data on all newly diagnosed malignancies since 1989, covering around 95% of the Dutch population [39]. The NCR is updated through PALGA and supplemented annually by information from hospital discharge records. Statistics Netherlands (CBS, www.cbs.nl)acted as a trusted third party to anonymize and link the data sets. The CBS used the date of birth, gender and six digit postal code, which were available in all three registries, to generate a unique personal identifier (Record Identification Number (RIN)). After the RIN was generated, all personally identifying data was removed from the data sets. The researchers used the RIN to link all three data sets. Ethical Approvals were given by the CMO Arnhem-Nijmegen, code: 2017-3912 in 18 December 2017. A waiver of informed consent was provided, no informed consent form was used.

### 4.2. Patient Selection and Variable Definitions

All patients aged ≥20 years of age with a diagnosed non-typhoidal *Salmonella* infection from the 1 January 1999, and with a diagnosed *Campylobacter* spp. infection from the 1 January 2002, until the 31 December 2016 were identified in the RIVM database. Additionally, all patients with non-intrahepatic biliary tract cancer (ICD-O-3 location codes C239, C240, C242, C243, C244, C248, C249) were identified in the NCR database. Patients who were diagnosed with intrahepatic BTC, BTC before or within 1 year of salmonellosis/campylobacteriosis diagnosis or had less than 1 year of follow-up were excluded. In case the patient had multiple recorded *Salmonella* spp./*Campylobacter* spp. infections, only the first diagnosis was considered. Both databases were cleared from duplicates. Time at risk was defined as the number of days between 1 year after salmonellosis/campylobacteriosis diagnosis and development of BTC, death, or end of the study period (31 December 2017), whichever occurred first.

### 4.3. Outcomes

The primary outcome of the study was the incidence of BTC among individuals with a registered non-typhoidal *Salmonella* or *Campylobacter* spp. infection in the past as compared to the incidence of BTC in the general Dutch population. Subgroup analyses were conducted to investigate the risk of BTC in patients ≤60 years of age (at the time of infection) and by gender.

### 4.4. Statistical Analysis

Standardized incidence ratios (SIR) were calculated for salmonellosis and campylobacteriosis patients separately to compare the difference in incidence of BTC in patients with *Salmonella* spp. or *Campylobacter* spp. infection to an age-, gender- and calendar year-matched cohort of the general Dutch population. To this end, the observed number of BTC cases in the salmonellosis and campylobacteriosis patients was divided by the expected number of BTC cases in the matched cohort provided by the NCR. 95% confidence intervals (95% CI) for the SIRs were calculated assuming a Poisson distribution. In all analyses, *p*-values < 0.05 were considered statistically significant. Statistical analysis was performed using STATA version 14 (StataCorp. 2015. Stata Statistical Software: Release 14. College Station, TX: StataCorp LP).

## 5. Conclusions

There is accumulating evidence that pathogenic bacteria like *Salmonella* spp. play a role in cancer development, including cancers of the digestive system. However, we could not demonstrate a significantly increased occurrence of BTC among reported salmonellosis or campylobacteriosis patients as compared to the general population. Potentially, the study was either underpowered due to the low number of BTC events or *Salmonella* and *Campylobacter* spp. infections are not associated with the development of BTC in Western countries. Additional research is needed to unravel the biological mechanisms behind bacterial infections as a cause of cancer and identify potential infections that may warrant early screening and therefore facilitate early cancer detection, especially in third-world countries with high rates of (hyper)endemic bacterial infections.

## Figures and Tables

**Table 1:** Baseline characteristics of the Salmonellosis and Campylobacterosis patients.

Characteristic	*N* (%)	Type
Age		Salmonellosis
<40	6167 (38%)	
40–49	2190 (13%)	
50–59	2445 (15%)	
60–69	2273 (14%)	
70–79	2003 (12%)	
80+	1172 (7%)	
Sex		
Male	7640 (47%)	
Female	8612 (53%)	
*Salmonella enterica* serotype		
*Typhimurium/monophasic var.*	4487 (28%)	
*Enteritidis*	5544 (34%)	
*(Para)Typhi*	318 (2%)	
Other serotypes and *S.* spp.	5903 (36%)	
Type of infection		
Septicemic	884 (5%)	
Enteric	13,864 (88%)	
Other	1066 (7%)	
Age		Campylobacterosis
<40	10,125 (37%)	
40–49	4499 (16%)	
50–59	4985 (18%)	
60–69	4153 (15%)	
70–79	2703 (10%)	
80+	1227 (4%)	
Sex		
Male	14,293 (52%)	
Female	13,399 (48%)	
*Campylobacter* species		
*C. jejuni*	23,647 (85%)	
*C. coli*	1910 (7%)	
Other species	2135 (8%)	
Type of infection		
Septicemic	^1^	
Enteric	^1^	
Other	^1^	

^1^ Not registered for campylobacterosis cases.

**Table 2:** Baseline characteristics of patients with BTC in the Netherlands (2000–2017).

Characteristic	*N* (%)/Median (95% CI)
Age	
<40	99 (1%)
40–49	326 (4%)
50–59	993 (12%)
60–69	2048 (24%)
70–79	2851 (33%)
80+	2189 (26%)
Sex	
Male	3822 (45%)
Female	4684 (55%)
Tumor location	
Gallbladder	2586 (30%)
Bile ducts, NOS	2421 (28%)
Proximal bile ducts	1846 (22%)
Distal bile ducts	1490 (18%)
Other ^1^	163 (2%)
Clinical stage	
Non-metastatic	4078 (48%)
Metastatic	4428 (52%)
Treatment	
Resection	2479 (29%)
Chemotherapy	647 (8%)
Survival (months)	5.8 (5.5–6.0)

^1^ Includes cystic duct and mixed types.

**Table 3:** Baseline characteristics of patients with *Salmonella* spp. infection and biliary tract cancer.

Characteristic	*N* (%)
Sex (male)	5 (56%)
Age	
<60	3 (33%)
≥60	6 (66%)
Serotype	
Enteritidis	4 (45%)
Typhimurium/monophasic var.	2 (22%)
Other	3 (33%)
Interval	
<60 months	7 (78%)
≥60 months	2 (22%)
Tumor location	
Gallbladder/proximal bile ducts	2 (22%)
Distal bile ducts	2 (22%)
Extrahepatic bile ducts, NOS	5 (56%)
Base of diagnosis	
Cytology/imaging	6 (67%)
Histology	3 (33%)

**Table 4:** Baseline characteristics of patients with *Campylobacter* spp. infection and biliary tract cancer.

Characteristic	*N* (%)
Sex (male)	3 (43%)
Age	
<60	2 (29%)
≥60	5 (71%)
Interval	
<60 months	3 (43%)
≥60 months	4 (57%)
Tumor location	
Gallbladder/proximal bile ducts	^1^
Distal bile ducts	^1^
Extrahepatic bile ducts, NOS	0 (0%)
Base of diagnosis	
Cytology/imaging	2 (29%)
Histology	5 (71%)

^1^ Numbers cannot be provided due to risk of subject identification.

**Table 5:** Incidence of biliary tract cancer in patients ≥1 year after laboratory-confirmed infection with *Salmonella* spp. or *Campylobacter* spp., stratified by age at infection and gender.

Type	Observed Incidence	Expected Incidence	SIR	95% CI	*p*-Value
***Salmonella* spp.**					
All patients	9	5.875	1.53	0.70–2.91	0.280
20–60	3	1.740	1.72	0.36–5.04	0.507
Male	5	2.665	1.88	0.61–4.38	0.264
Female	4	3.289	1.22	0.33–3.11	0.835
***Campylobacter* spp.**					
All patients	7	7.221	0.97	0.39–2.00	0.868
20–60	2	2.126	0.94	0.11–3.40	0.715
Male	3	4.025	0.75	0.15–2.18	0.857
Female	4	3.233	1.24	0.34–3.17	0.810

## References

[1] Surveillance EaERp. Biliary Tract Cancer, Survival by Stage 2018. https://www.cancer.org/cancer/bile-duct-cancer/detection-diagnosis-staging/survival-by-stage.html.

[2] Ries L.A.G., Reichman M.E., Lewis D.R., Hankey B.F., Edwards B.K. (2003). Cancer Survival and Incidence from the Surveillance, Epidemiology, and End Results (SEER) Program. Oncologist.

[3] Cronin K.A., Bs A.J.L., Scott S., Sherman R.L., Noone A.-M., Ms N.H., Henley S.J., Anderson R.N., Bs A.U.F., Ma J. (2018). Annual Report to the Nation on the Status of Cancer, part I: National cancer statistics. Cancer.

[4] Shroff R.T., Kennedy E.B., Bachini M., Bekaii-Saab T., Crane C., Edeline J., El-Khoueiry A., Feng M., Katz M.H., Primrose J. (2019). Adjuvant Therapy for Resected Biliary Tract Cancer: ASCO Clinical Practice Guideline. J. Clin. Oncol..

[5] Public Health Agency of Canada, Statistics Canada, Canadian Cancer Society (2019). Release notice-Canadian Cancer Statistics 2019. Health Promot. Chronic Dis. Prev. Can..

[6] Are C., Ahmad H., Ravipati A., Croo D., Clarey D., Smith L., Price R.R., Butte J.M., Gupta S., Chaturvedi A. (2017). Global epidemiological trends and variations in the burden of gallbladder cancer. J. Surg. Oncol..

[7] Lohman E.D.S., De Bitter T., Verhoeven R.H., Van Der Geest L., Hagendoorn J., Mohammad N.H., Daams F., Klümpen H.-J., Van Gulik T., Erdmann J.I. (2020). Trends in Treatment and Survival of Gallbladder Cancer in the Netherlands; Identifying Gaps and Opportunities from a Nation-Wide Cohort. Cancers.

[8] Tyson G.L., El-Serag H.B. (2011). Risk factors for cholangiocarcinoma. Hepatology.

[9] Gatto M., Bragazzi M.C., Semeraro R., Napoli C., Gentile R., Torrice A., Gaudio E., Alvaro D. (2010). Cholangiocarcinoma: Update and future perspectives. Dig. Liver Dis..

[10] Schottenfeld D., Beebe-Dimmer J. (2015). The cancer burden attributable to biologic agents. Ann. Epidemiol..

[11] Wardak S. (2016). Human Papillomavirus (HPV) and cervical cancer. Medycyna Doswiadczalna i Mikrobiologia.

[12] Balogh J., Victor D., Asham E.H., Burroughs S.G., Boktour M., Saharia A., Li X., Ghobrial R.M., Monsour H.P. (2016). Hepatocellular carcinoma: A review. J. Hepatocell. Carcinoma.

[13] Aitken C.A., Van Agt H.M.E., Siebers A.G., Van Kemenade F.J., Niesters H.G.M., Melchers W.J.G., Vedder J.E.M., Schuurman R., Brule A.J.C.V.D., Van Der Linden H.C. (2019). Introduction of primary screening using high-risk HPV DNA detection in the Dutch cervical cancer screening programme: A population-based cohort study. BMC Med..

[14] Gagnaire A., Nadel B., Raoult D., Neefjes J., Gorvel A.G.B.N.J.-P. (2017). Collateral damage: Insights into bacterial mechanisms that predispose host cells to cancer. Nat. Rev. Genet..

[15] Samaras V., Rafailidis P.I., Mourtzoukou E.G., Peppas G., Falagas M.E. (2010). Chronic bacterial and parasitic infections and cancer: A review. J. Infect. Dev. Countries.

[16] Scanu T., Spaapen R.M., Bakker J.M., Pratap C.B., Wu L.-E., Hofland I., Broeks A., Shukla V.K., Kumar M., Janssen H. (2015). *Salmonella* Manipulation of Host Signaling Pathways Provokes Cellular Transformation Associated with Gallbladder Carcinoma. Cell Host Microbe.

[17] Mughini-Gras L., Schaapveld M., Kramers J., Mooij S., Neefjes-Borst E.A., Van Pelt W., Neefjes J. (2018). Increased colon cancer risk after severe *Salmonella* infection. PLoS ONE.

[18] Brauner A., Brandt L., Frisan T., Thelestam M., Ekbom A. (2010). Is there a risk of cancer development after Campylobacter infection?. Scand. J. Gastroenterol..

[19] Lara-Tejero M. (2001). A Bacterial Toxin that Causes DNA Damage to Modulate Cellular Responses. Sci. World J..

[20] He Z., Gharaibeh R.Z., Newsome R.C., Pope J.L., Dougherty M.W., Tomkovich S., Pons B., Mirey G., Vignard J., Hendrixson D.R. (2018). *Campylobacter jejuni* promotes colorectal tumorigenesis through the action of cytolethal distending toxin. Gut.

[21] Wu N., Yang X., Zhang R., Li J., Xiao X., Hu Y., Chen Y., Yang F., Lu N., Wang Z. (2013). Dysbiosis Signature of Fecal Microbiota in Colorectal Cancer Patients. Microb. Ecol..

[22] Marcano-Bonilla L., Mohamed E.A., Mounajjed T., Roberts L.R. (2016). Biliary tract cancers: Epidemiology, molecular pathogenesis and genetic risk associations. Chin. Clin. Oncol..

[23] Caygill C., Hill M., Braddick M., Sharp J. (1994). Cancer mortality in chronic typhoid and paratyphoid carriers. Lancet.

[24] Avilés-Jiménez F., Guitron A., Segura-López F., Méndez-Tenorio A., Iwai S., Hernández-Guerrero A., Torres J. (2016). Microbiota studies in the bile duct strongly suggest a role for Helicobacter pylori in extrahepatic cholangiocarcinoma. Clin. Microbiol. Infect..

[25] Edwards S., Lilford R.J., Braunholtz D., Jackson J. (1997). Why “underpowered” trials are not necessarily unethical. Lancet.

[26] Nagaraja V., Eslick G.D. (2014). Systematic review with meta-analysis: The relationship between chronic *Salmonella typhi* carrier status and gall-bladder cancer. Aliment. Pharmacol. Ther..

[27] Safaeian M., Gao Y.-T., Sakoda L.C., Quraishi S.M., Rashid A., Wang B.-S., Chen J., Pruckler J., Mintz E., Hsing A.W. (2011). Chronic typhoid infection and the risk of biliary tract cancer and stones in Shanghai, China. Infect. Agents Cancer.

[28] Iyer P., Barreto S.G., Sahoo B., Chandrani P., Ramadwar M.R., Shrikhande S.V., Dutt A. (2016). Non-typhoidal *Salmonella* DNA traces in gallbladder cancer. Infect. Agents Cancer.

[29] Egal-Mor O., Boyle E.C., Grassl G.A. (2014). Same species, different diseases: How and why typhoidal and non-typhoidal *Salmonella enterica* serovars differ. Front. Microbiol..

[30] Integraal Kankercentrum Nederland: Cijfers over Kanker. https://www.iknl.nl/nkr-cijfers.

[31] Huai J.-P., Ding J., Ye X.-H., Chen Y.-P. (2014). Inflammatory bowel disease and risk of cholangiocarcinoma: Evidence from a meta-analysis of population-based studies. Asian Pac. J. Cancer Prev..

[32] Massa A., Varamo C., Vita F., Tavolari S., Peraldo-Neia C., Brandi G., Rizzo A., Cavalloni G., Aglietta M. (2020). Evolution of the Experimental Models of Cholangiocarcinoma. Cancers.

[33] Rizzo A., Ricci A.D., Tober N., Nigro M.C., Mosca M., Palloni A., Abbati F., Frega G., De Lorenzo S., Tavolari S. (2020). Second-line Treatment in Advanced Biliary Tract Cancer: Today and Tomorrow. Anticancer Res..

[34] Havelaar A.H., Ivarsson S., Löfdahl M., Nauta M. (2012). Estimating the true incidence of campylobacteriosis and salmonellosis in the European Union, 2009. Epidemiol. Infect..

[35] Friesema I., Koppeschaar C., Donker G., Dijkstra F., Van Noort S., Smallenburg R., Van Der Hoek W., Van Der Sande M. (2009). Internet-based monitoring of influenza-like illness in the general population: Experience of five influenza seasons in the Netherlands. Vaccine.

[36] A Iacobuzio-Donahue C. (2012). Genetic evolution of pancreatic cancer: Lessons learnt from the pancreatic cancer genome sequencing project. Gut.

[37] Van Pelt W., De Wit M.A.S., Wannet W.J.B., Ligtvoet E.J.J., Widdowson M.A., Van Duynhoven Y.T.H.P. (2003). Laboratory surveillance of bacterial gastroenteric pathogens in The Netherlands, 1991–2001. Epidemiol. Infect..

[38] Casparie M., Tiebosch A.T.M.G., Burger G., Blauwgeers H., Van De Pol A., Van Krieken J.H.J.M., A Meijer G. (2007). Pathology Databanking and Biobanking in The Netherlands, a Central Role for PALGA, the Nationwide Histopathology and Cytopathology Data Network and Archive. Cell. Oncol..

[39] Van Der Willik K.D., Ruiter R., Van Rooij F.J., Heemst J.V., Hogewoning S.J., Timmermans K.C., Visser O., Schagen S.B., Ikram M.A., Stricker B.H. (2019). Ascertainment of cancer in longitudinal research: The concordance between the Rotterdam Study and the Netherlands Cancer Registry. Int. J. Cancer.

